# Spatio-Temporal Variability of the North Sea Cod Recruitment in Relation to Temperature and Zooplankton

**DOI:** 10.1371/journal.pone.0088447

**Published:** 2014-02-13

**Authors:** Delphine Nicolas, Sébastien Rochette, Marcos Llope, Priscilla Licandro

**Affiliations:** 1 Sir Alister Hardy Foundation for Ocean Science, Plymouth, Devon, United Kingdom; 2 Department of Coastal Environment Dynamics, Ifremer, Plouzané, France; 3 Instituto Español de Oceanografía, Centro Oceanográfico de Cádiz, Cádiz, Andalucía, Spain; Institut Maurice-Lamontagne, Canada

## Abstract

The North Sea cod (*Gadus morhua*, L.) stock has continuously declined over the past four decades linked with overfishing and climate change. Changes in stock structure due to overfishing have made the stock largely dependent on its recruitment success, which greatly relies on environmental conditions. Here we focus on the spatio-temporal variability of cod recruitment in an effort to detect changes during the critical early life stages. Using International Bottom Trawl Survey (IBTS) data from 1974 to 2011, a major spatio-temporal change in the distribution of cod recruits was identified in the late 1990s, characterized by a pronounced decrease in the central and southeastern North Sea stock. Other minor spatial changes were also recorded in the mid-1980s and early 1990s. We tested whether the observed changes in recruits distribution could be related with direct (i.e. temperature) and/or indirect (i.e. changes in the quantity and quality of zooplankton prey) effects of climate variability. The analyses were based on spatially-resolved time series, i.e. sea surface temperature (SST) from the Hadley Center and zooplankton records from the Continuous Plankton Recorder Survey. We showed that spring SST increase was the main driver for the most recent decrease in cod recruitment. The late 1990s were also characterized by relatively low total zooplankton biomass, particularly of energy-rich zooplankton such as the copepod *Calanus finmarchicus*, which have further contributed to the decline of North Sea cod recruitment. Long-term spatially-resolved observations were used to produce regional distribution models that could further be used to predict the abundance of North Sea cod recruits based on temperature and zooplankton food availability.

## Introduction

Changes in the spatial distribution of species over time reflect variations in the suitability of both biotic and abiotic environmental conditions regarding their survival, reproduction or dispersion [1]. In the North Sea, Atlantic cod (*Gadus morhua*, L.) has severely declined over the past four decades [2], [3], while sea surface temperature (SST) has increased [4], [5]. The North Sea is at the south-easternmost edge of Atlantic cod distribution. In this region, where cod has been heavily overfished since the late 1960s [2], [3], the stock has now a reduced reproductive capacity (i.e. truncated age structure and reduced spawning stock biomass, SSB), and is likely to be less resilient to the effects of environmental changes [2], [6], [7]. Indeed, the fluctuations of a stock with a reduced age composition largely depend on recruitment success, which in turn may be strongly affected by different environmental factors [8]–[11]. Hence, both overfishing and climate change are considered to be the main drivers of the drastic decrease of North Sea cod. Disentangling their relative impacts remains challenging [6].

Multiple internal (e.g. egg quality) and external factors affect the mortality of early life stages and hence recruitment variability [12]. Temperature and food availability are two key environmental factors that regulate cod recruitment success, affecting the timing and production of eggs during winter [13]–[16] and the survival of pelagic larvae during spring [17]–[20]. The rise of temperature above the species optimum has a negative impact on cod larval development [21]–[23]. Although warmer temperatures can be associated with a faster growth, they might also determine higher larval metabolic requirements [24], [25], while reducing the availability of cod’s favourite planktonic prey [17]. Changes in the timing of plankton production associated with increasing temperatures may also result in a mismatch between cod larvae and suitable planktonic prey [26]. Accordingly, North Sea cod recruitment is expected to be more successful during relatively cold years than during warm ones [22], [27], [28].

Beaugrand et al. [29] suggested that temperature affects the recruitment of North Sea cod mainly indirectly via its plankton prey. Subsequently, Olsen et al. [28] provided evidence for a combined direct and indirect effect of temperature on cod larvae. From the 1960s to the mid-1980s, a period identified as a ‘gadoid outburst’ [30], the successful recruitment of cod coincided with cold conditions, favourable seasonal timing and high abundance of cod’s favoured prey [17]. Specifically, the survival of cod larvae was positively related to high abundance of calanoid copepods, in particular *Calanus finmarchicus* and *Pseudocalanus* spp., and of euphausiids [17]. As temperature increased over the years, the cold-temperate *C. finmarchicus* has declined, while the warm-temperate *Calanus helgolandicus* has increased [31], [32]. Accordingly, the annual peak of total *Calanus* abundance shifted from spring to late summer, when juvenile cod have usually already switched to feed primarily upon euphausiids and fish larvae [17], [33]. Together with a decline of the size and quantity of plankton prey, this mismatch had been associated with the persistent poor recruitment of cod since the mid-1980s [17], [23].

Between 1982 and 1988, the North Sea ecosystem has gone through a stepwise change, from phytoplankton to zooplankton and fish [34]. In that period the position of the 9–10°C isotherm (SST annual average) has moved northwards [35]. The thermal boundary of 9–10°C, which represents a critical threshold separating different North Atlantic ecosystems (i.e. the Atlantic Polar biome and the Atlantic Westerly Winds biome [36], [37]), corresponds to the southern edge of the spatial distribution of North Atlantic cod [35]. Indeed, annual SST lower than 10°C and high abundance of energy-rich planktonic prey species such as the large copepod *C. finmarchicus* appear to be two determining factors of the cod’s ecological niche [29].

The present study integrates all available sources of long-term information with high spatial resolution to provide a comprehensive spatially-explicit analysis of how temperature-driven processes have impacted recruitment and population dynamics of North Sea cod. The bottom-up regulation of cod recruitment in the North Sea has been previously analysed using synthetic indices of long time series [17], [29], [35], while spatially-explicit models were only explored on a few pre-selected years [26]. The present study avoids the use of composites and focuses instead on spatially-referenced biomasses of cod prey items in order to determine their specific role.

To explore the variability of cod recruit density in space over the past decades, we analyzed data collected from 1974 to 2011 using a statistical approach based on generalized additive models (GAM) or polynomial linear models (LM) tailored to detect non-additive dynamics. Our model was designed to identify turnover years marking significant differences between periods of common spatio-temporal distribution (threshold model). This methodology stemmed from Ciannelli et al. [38], [39] who separately applied threshold GAMs and spatial GAMs to fish populations. In the present study both types of GAMs were combined as in Llope et al. [40] and the level of uncertainty associated with each year of change was assessed. This new development enables assessing the speed of change between periods and the importance of turning point years. Based on this method, we at first identified the major changes in the spatio-temporal distribution of the cod population (i.e. recruits and adults). Then, we tested whether the observed changes in the distribution of cod recruits could be related to direct (i.e. change in temperature) or indirect (i.e. change in fish SSB, quantity and quality of planktonic prey) effects of climate change. Finally, we produced different distribution models, validated against long-term spatially-resolved observations that could further be used to predict the abundance of cod recruits in distinct North Sea regions, based on temperature and zooplankton food availability.

## Materials and Methods

### Data

#### Fish data

Data on cod adults and juveniles collected until 2011 in the North Sea (between latitudes of 51°N and 61°N) during the International Bottom Trawl Survey (IBTS) were obtained from the DATRAS (DAtabase TRAwl Surveys) database operated by the International Council for the Exploration of the Sea (ICES, http://ecosystemdata.ices.dk/). To allow a good spatial cover of the studied area, the time series taken into account covered the period 1974–2011. IBTS surveys were carried out at least two hauls per ICES statistical rectangles of 1°Lat *0.5°Long per quarter of the year. Each haul was characterised by a longitude (*λ*), a latitude (*Φ*), a year (*y*) and a Catch Per Unit Effort (CPUE). Longitude was corrected to transform decimal degrees of longitude into decimal degrees of latitude that are constant distance using Mercator-type projection formula (λ*cos([Φ*π]/180)).

**Figure 1:**
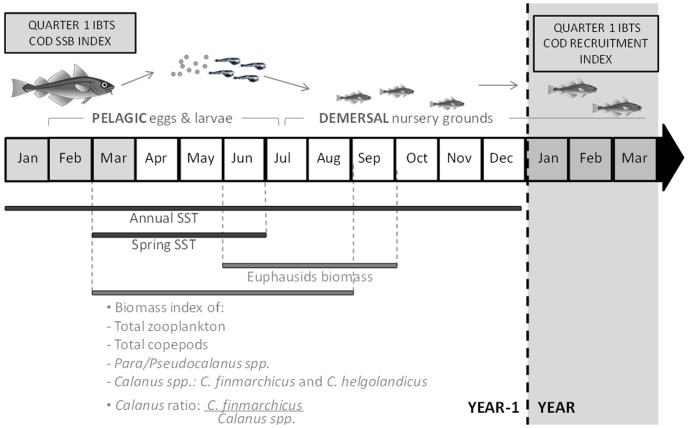
Environmental descriptors and cod recruitment. Summary of descriptors of cod population (i.e. Q1 IBTS cod recruitment index and Q1 IBTS cod SSB index) and of North Sea environment (i.e. SST and zooplankton biomass indices) considered in the present study. Note that each environmental descriptor has been averaged over different periods of the year, according to the different phases of North Sea cod life cycle.

In the North Sea, cod usually spawn from February to May with a peak of egg production in March [41], [42]. Pelagic eggs hatch after a few weeks and the pelagic phase lasts until July-August, when juveniles switch to a demersal life style [43]. The density of age-1 recruits in the first quarter of the year (Q1) was used as an index of cod recruitment in the previous year (Fig. 1). As Atlantic cod tend to mature at 4 years old [44], the density of cod aged 4 years or more in Q1 was used as an index of the spawning stock biomass [45].

#### Temperature data

Monthly SST (°C) data (1930–2010) on a 1*1 degree area grid were obtained from the British Atmospheric Data Center (BADC) HadISST 1.1 dataset (http://badc.nerc.ac.uk/home/). Both annual and spring (March to June) SST were retained as those periods have previously proved to be good proxies for environmental dynamics affecting cod recruitment [18], [35].

#### Zooplankton data

Zooplankton biomass (Dry Weight) was derived from Continuous Plankton Recorder (CPR) records by multiplying the abundance of each species by the average dry weight per individual obtained either by direct measurements or calculated using dry weight species-specific weight-length regression equations (see [40] for more details). A detailed description of the CPR sampling and analysis can be found in Richardson et al. [46]. The zooplankton data set analysed consisted of a total of 28940 samples collected in the North Sea (51°N to 61°N/5°W to 9°E) from March to September 1958–2009. As routes of ships varied over the years, data were interpolated over a regular grid having an equivalent distance of 1*1 decimal degree of latitude (111 km*111 km) using inverse distance interpolation with a radius of 50 km [47], [48].

From early larvae to juvenile stages the diet of cod recruits gradually changes from nauplii and copepodite stages of copepods (April-May), to adult copepods (June-July) and euphausiids (from August onwards) [33], [49], [50]. *Pseudocalanus elongatus* together with *C. finmarchicus* are considered among the dominant prey items for cod larvae [49], [50]. Considering the changes in the diet of cod early life stages and the taxa routinely identified in CPR samples, eight biomass (dry weight) indices were tested: from March to August, (1) total zooplankton and copepods biomass as an index of total food availability; (2) biomass of the calanoid copepods *Para/Pseudocalanus* spp. (accounting for both juvenile and adult stages of *Pseudocalanus* and *Paracalanus* spp.), *C. finmarchicus* and *C. helgolandicus*, (3) total *Calanus* biomass, i.e. the combination of the two previous *Calanus* species, (4) ratio between the two *Calanus* spp. (*C. finmarchicus* biomass/[total *Calanus* spp. biomass]) to represent the changes in the *Calanus* community; and from June to September (5) total euphausiids biomass (Fig. 1).

### Identification of Turnover Year(s) Based on Threshold Spatially-explicit Models

We used an approach based on generalised additive models and polynomial linear models to select turnover years (y*) identifying a change between periods of similar spatio-temporal distributions. GAMs were tested to fit the shapes of distributions. Modelling the spatial distribution of species using latitude and longitude as parameters requires models allowing for non-linearities. GAMs, due to their flexibility, met this requirement, but the relatively low number of observations in space suggested testing more robust parametric methods like LM. Using polynomial LM allowed for fitting the non-linearity of spatial distributions. A validation step allowed a choice between the two approaches.

Different model formulations were tested separately on (i) log-transformed CPUE, (ii) log-transformed biomass of different plankton species, (iii) SST and (iv) *Calanus* ratio. The variable to explain, log-transformed CPUE (log CPUE +1), is noted *X_(λ,Φ,y)_* in the following, with *λ* the longitude, *Φ* the latitude and *y* the year. The final combination of turnover year(s) was defined by the number of thresholds (tested from 0 to 4) that ensured a minimum of 2 year periods. For each (or combination of) possible threshold year(s), different GAM and polynomial LM models were fitted. The formulation of GAM models is:
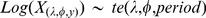
(1)where the tensor function (*te*) includes the single and the interaction effects of latitude and longitude and where *period* represents the index of the period between two identified turnover years, accounted as a factor.

The formulation of polynomial LM models is:
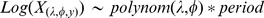
(2)


#### Model Selection

To interpolate the spatial distribution with parsimony, the smooth parameter of the tensor of the GAM or the maximum degree of the polynomial in LM was limited to a maximum *k*. The choice of the *k* parameter was based on a bootstrap cross-validation method. The maximum *k* was increased until the gain within the following bootstrap validation method was no longer significant. All combination of periods based on different threshold years were also tested.

The model selection was thus based on a cross-validation method coupled to a bootstrap. The Akaike criterion (AIC) is a measure of the relative quality of a statistical model that takes into account the goodness of fit and complexity of a given model. As such, AIC provides a means for model selection [51], the lower the AIC, the better the model. However, it can sometimes overestimate the amount of parameters needed [52]. Hence, for each combination of thresholds and each degree (in tensor or polynomial terms) tested (from 0 to 4), models were fitted on a random sub-sample containing 75% of the data. The model was then used to predict log-CPUE of the 25% sub-sample left. This was repeated 50 times on different random sub-samples as this was a good compromise between computing time and convergence of the results. The adjusted explained deviance was calculated (*D^2^*) on the validation sub-sample [53] as an index of the goodness of fit of the models:
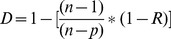
(3)

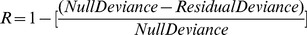
(4)where *n* is the number of observations (in the 25% sub-sample) and *p* the number of degrees of freedom used to fit the model. The higher the *D^2^*, the better the model. The AIC and the *D^2^* were estimated and stored for each sub-sample. The distributions of these parameters over the 50 replicates were then compared (with a t-student test means comparison) to select the most parsimonious model having the best results in explaining the spatio-temporal distribution. This method also permitted the selection of the best amount and combination of threshold years, thus periods, if any. Once selected, the best threshold model was run with the entire dataset for graphical representation. Models predictions were estimated for a regular grid to map the spatial pattern of each period between threshold years.

AIC comparison between equivalent LM and GAM formulations suggested that LMs were more parsimonious. When comparing the adjusted explained deviance (*D^2^*) on the validation sub-samples, LM models explained 5 to 10% more of explained deviance than GAM models. Therefore, spatial distributions shown in results were issued from the polynomial LM models.

#### Estimation of uncertainty on threshold years

The method allowed the identification and ordering of the turnover years by importance and the assessment of the speed of the change between periods. The best model in terms of amount of degrees and of thresholds was run while adding thresholds one by one. The gain of explained deviance was calculated for each step from no threshold to the total number of selected thresholds, each year being tested as the potential threshold. Once the best year was chosen, the following step calculated the gain in explained deviance while using each of the remaining years as a second threshold, and so on. The maximum gain in explained deviance accounted for the importance of each threshold. The difference in gain of deviance associated with years around a specific threshold was used to assess the speed of change between periods.

#### Approximation of the inter-annual variability and graphical representation for comparison with explanatory variables

For graphical representation of raw data, the variables of interest were interpolated inter-annually on a regular prediction grid of 0.5*0.5° latitude for cod, 1*1° for SST and zooplankton. For each year and each node of the grid, raw log-CPUE (or log-biomass or SST) were estimated by a simple inverse distance interpolation method [48] within a 50 km radius on the raw data. Averaged environmental parameters were estimated for each notch of the grid in the periods identified by the best model for cod recruits, taking into account a lag time of one year (e.g. 1973–1983 for the cod period 1974–1984).

### Environmental Effects on Cod Recruitment

**Figure 2:**
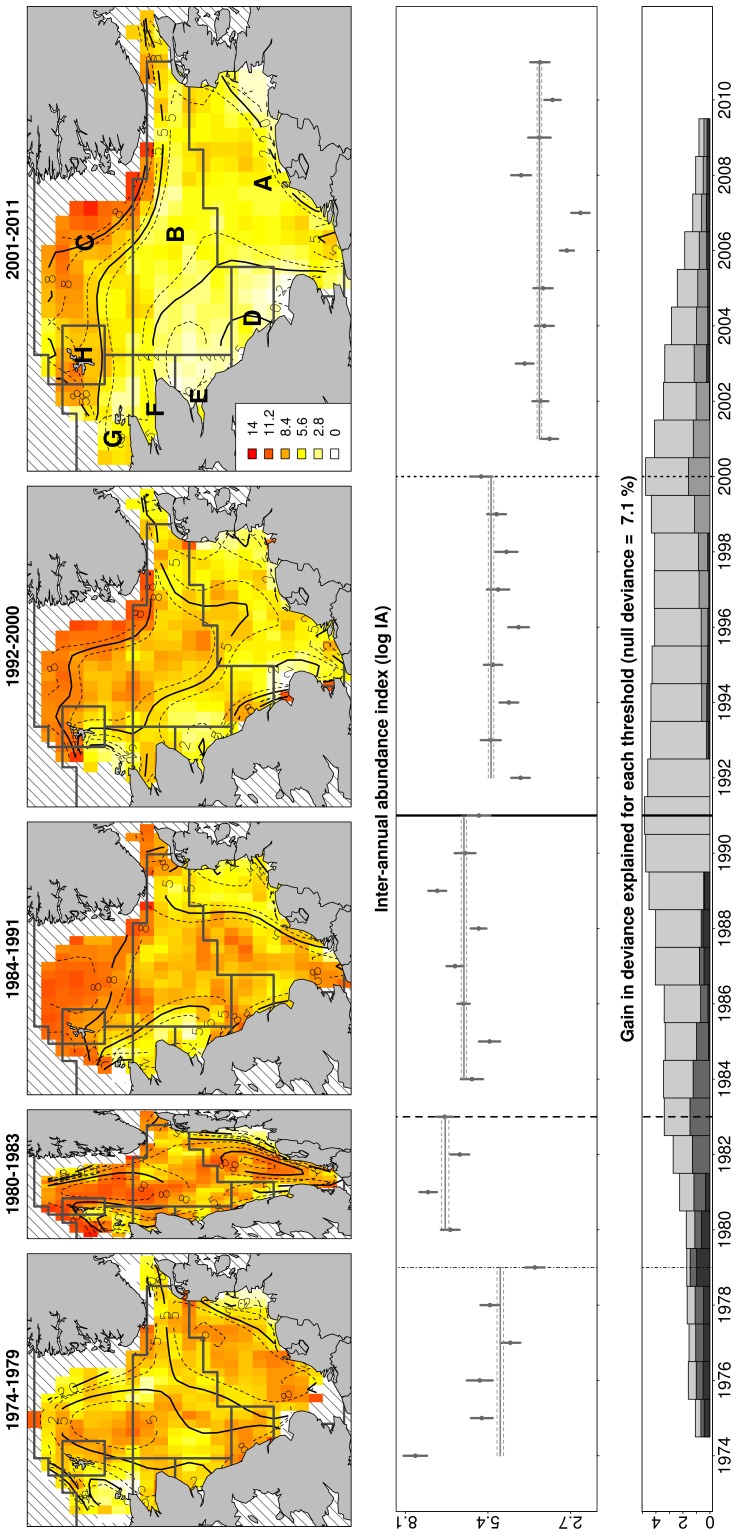
Spatio-temporal variability of cod age-4 or older. Identification of main changes in the spatio-temporal variability of cod age-4 or older between 1974 and 2011, based on ICES data collected during the first quarter of the year. First panel (a): maps of mean cod density distribution per identified period (lines represents 95% of uncertainty on predictions). Polygons, i.e. regions corresponding to different cod sub-populations, are superimposed. Second panel (b): Inter-annual variability of the abundance index (mean and standard deviation per year). Third panel (c): Uncertainty around each threshold year: gain in explained deviance per year, around the years of highest (light grey) or lower but still significant (medium to dark grey) changes in distribution.

**Figure 3:**
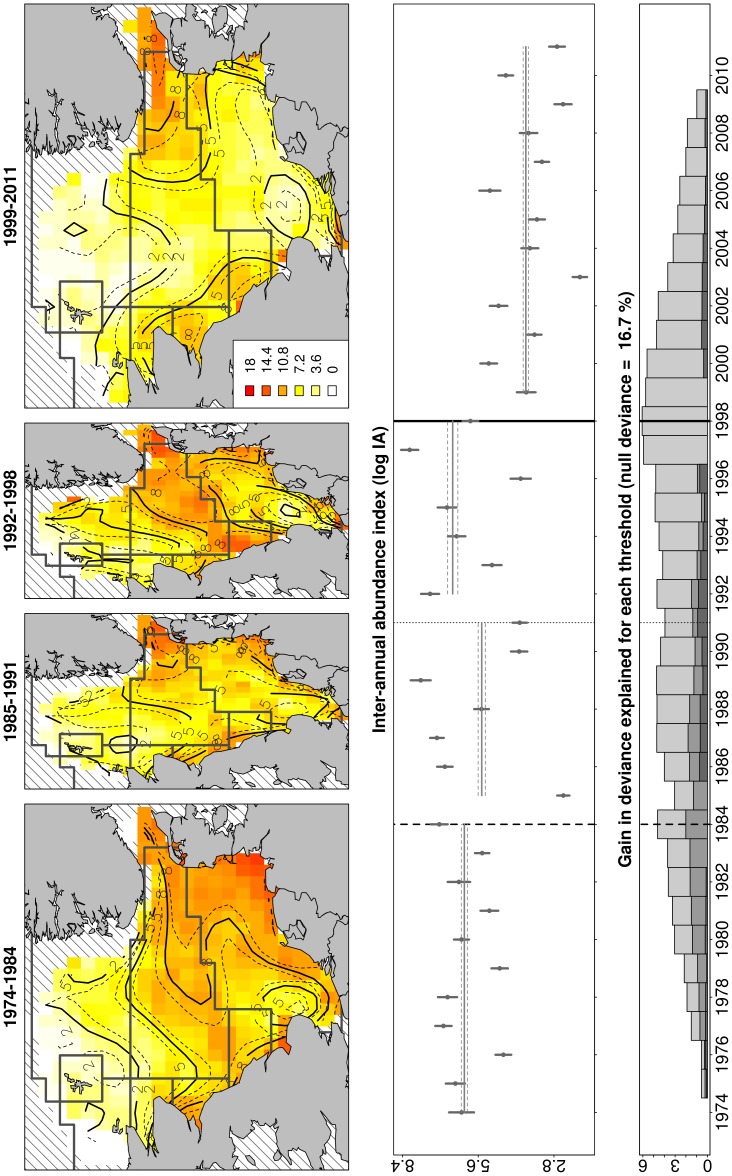
Spatio-temporal variability of cod age-1. Identification of main changes in the spatio-temporal variability of cod age-1 between 1974 and 2011 based on ICES data during the first quarter of the year. Panels are described in the caption of figure 2.

From spawning to recruitment in nursery grounds, cod pelagic stages may migrate fairly long distances, experiencing different conditions of temperature and food availability. This extended drift made it meaningless to directly relate for each notch of the grid the explanatory environmental variables with the cod recruitment index. Previous studies have suggested that North Sea cod is composed of several genetically distinct subpopulations [54]–[56]. We considered the regions defined by Heath et al. [56] to test the relationship between cod recruitment and environmental variables (Fig. 2a). Heath et al.’s [56] regions (from here onwards called ‘polygons’) delineate eight different cod subpopulations (named regions A to H, Fig. 2a). In each polygon, the spawning and nursery grounds of the associated subpopulation are located. Only the gridded values of the recruitment indices corresponding to the nursery ground areas within each polygon (Fig. 3a) were finally selected for models. The assumption was that cod recruits caught in a nursery area of one polygon have been under the influence of environmental conditions in the same polygon during their first year of growth.

#### Direct effect of SSB and environment on recruitment

Correlations between variables (VAR) were first analysed using Spearman rank correlation. Different models tested separately the effect of cod SSB, SST (i.e. annual and spring temperatures) and zooplankton indices on recruitment variability (*log(CPUE_age1_+1)*) accounting for (i) all the eight polygons and (ii) only for the central and southern polygons (i.e. A, B, D and E, Fig. 2a), where the main nurseries of North Sea cod are located and where zooplankton data were well spatially represented. GAMs and polynomial linear models were used to test the correlations. The formulation of polynomial LM models is:
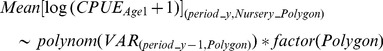
(5)where the periods *(period_y-1)* used for the explanatory variables corresponds to the cod recruitment periods *(period_y)* with a lag time of one year (y-1). GAMs were also tested using the same equation except that polynom was replaced by a tensor. A cross-validation coupled with a bootstrap was run to compare the effects of different tested variables. The deviance explained by the different environmental descriptors for each of the validation subsamples was calculated and a student test of mean comparison helped finding the model having the highest explained deviance.

#### Indirect effect of SST on recruitment

Temperature may indirectly affect fish recruitment, influencing the adult stock biomass and the prey availability. Once the periods characterised by different spatio-temporal distribution were identified, we tested the effect of SST changes on cod SSB of the following year:
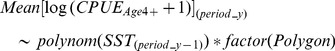
(6)


In an attempt to disentangle the effect of temperature and fishing mortality on the variability of cod adult stock, the ICES *Fbar* index (exp(-*Fbar*)), which assesses the fishing pressure applied on age-2–4 cod [3], was tested against SST. However, this only provided a rough indication of the effect of overfishing on cod SSB as the *Fbar* index is not spatially resolved and represents an annual average for the whole North Sea, Skagerrak and English Channel regions. This was not further explored.

The indirect effect of temperature on zooplankton prey was tested by considering the periods identified by the analysis of cod recruitment variability:
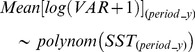
(7)


A set of models with different combinations of variables were tested using the periods identified by the analysis of cod recruitment variability. An attempt was made to try to disentangle direct and indirect effects of temperature on recruitment, as often the first tends to override the second. The final model of cod recruitment was built using a stepwise procedure. At first, cod SSB was selected as 1^st^ covariate, based on the assumption that the number of recruits is dependent on the size of the adult stock; then the most relevant zooplankton descriptor/s was/were selected according to the associated gain in explained deviance. Finally, temperature was included as a covariate if its contribution to the residual deviance was still significant. Models were tested with and without the multiplicative Polygon effect and the interaction effect between temperature and other environmental variables.

All models assumed a Gaussian distribution of the residuals, except for *Calanus* ratio, which required a binomial model. All analyses were run using the R software [57] and the ‘*mgcv*’ package for the GAM models [58].

## Results

### Spatio-temporal Changes in the Distribution of the North Sea Cod Population

From 1974 to 2011, the centre of distribution of adult (i.e. age-4^+^) cod moved northwards, outside the North Sea. Adult cod started to decrease in the southeastern North Sea and offshore Scotland (Fig. 2a), the main changes occurring around 1991 and 2000, the years associated respectively with the first and second most significant gains in deviance (Fig. 2c). The gain function was consistently high for all the years between 1989 and 2001 suggesting that the depletion of the North Sea adult cod was a gradual process spanning over a decade. Since 2001, adult cod stock was very low in the whole southern and central North Sea, as compared with the Norwegian trench and the Shetland Islands area (on average 2.8±0.2 log CPUE in polygons A, B, D and E vs 6.4±0.4 in polygons C and H, Fig. 2a).

From 1974 until the late 1990s, North Sea cod recruits (i.e. one year old) were relatively abundant (Fig. 3b) and mainly concentrated in the shallow sheltered nursery areas along the British and European continental coastlines and around Dogger Bank (Fig. 3a). From the mid-1980s to the late 1990s, cod recruitment was highly variable (Fig. 3b), with the core distribution of the juveniles mainly located around the Skagerrak, Flamborough Head and the Central North Sea. The year 1998 marked the most important spatio-temporal change in their distribution, with a significant depletion of cod juveniles recorded in most North Sea regions (Fig. 3a,b). This major change, based on the gain function, appeared less gradual when compared to change that occurred in the adult stock (Fig. 2c). Since 1998, cod recruits oscillated around 4.8 log CPUE (Fig. 3b), about only 2/3 of the average observed during 1974–1984 (7.6±0.4 log CPUE), and were mainly found along the British coast and in the Skagerrak.

### Links between Environment and Main Spatio-temporal Changes in Cod Recruitment

**Figure 4:**
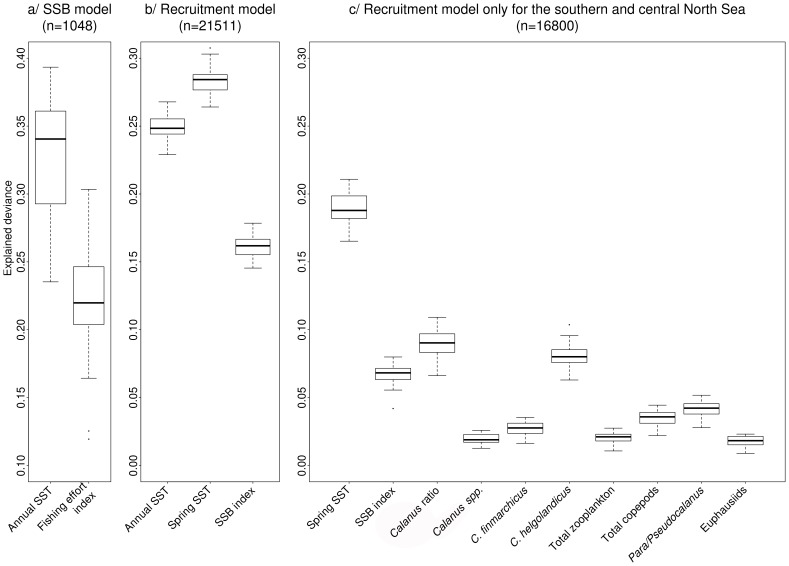
Deviance explained by environmental descriptors. Box and whisker plots comparing the deviance explained by different environmental descriptors in three different models of cod variability. (a) Model describing the variability of the size of cod adult stock, (b) recruitment model for the whole North Sea, (c) recruitment model for the central and southern North Sea (c). The effect of fishing pressure on the size of cod adult stock was also tested.

**Figure 5:**
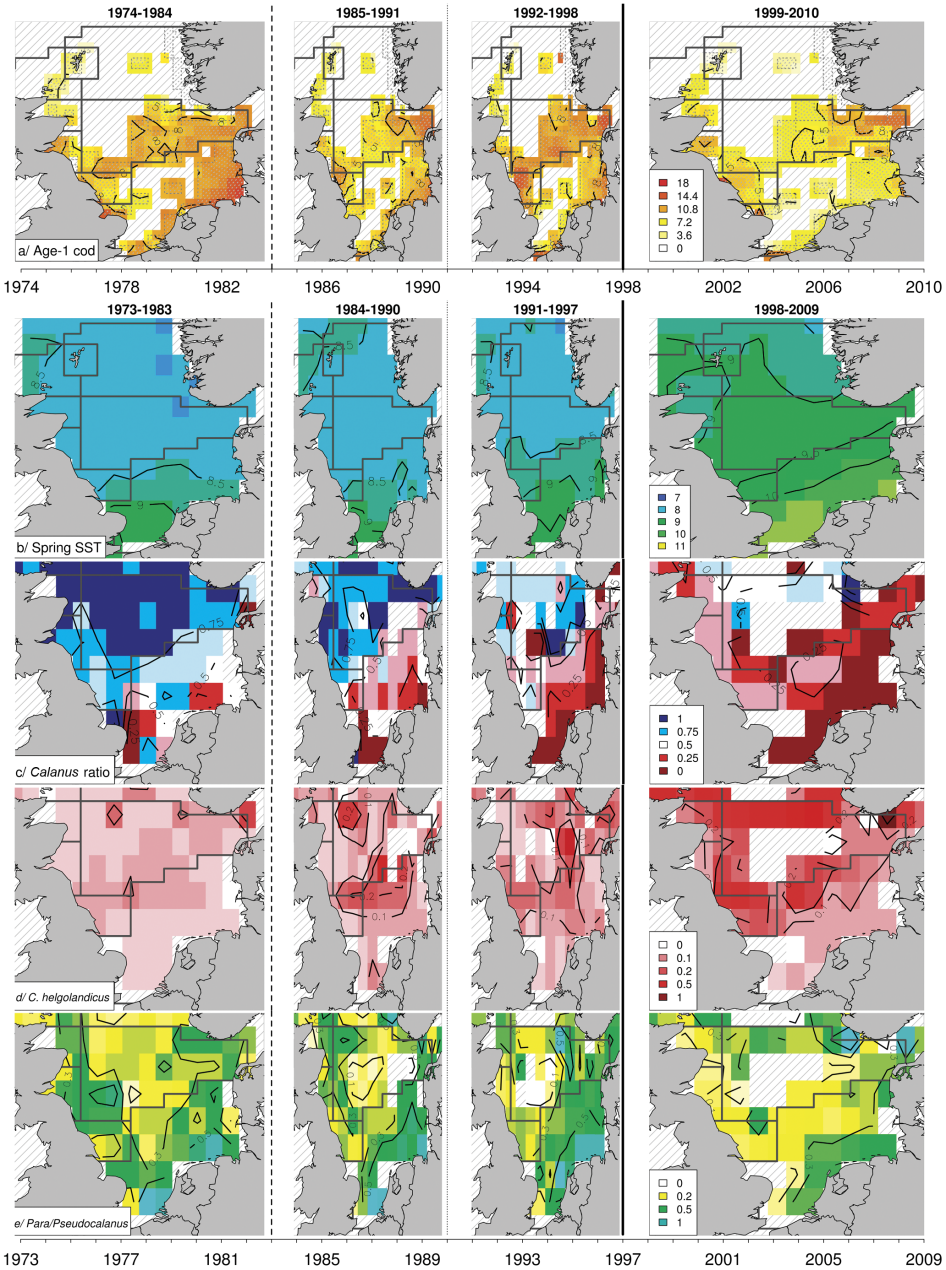
Spatio-temporal distributions of cod recruits and of most relevant environmental factors. (a) Main changes in spatio-temporal distribution of cod recruits (logCPUE) between 1974 and 2010 within North Sea nursery areas (as identified by [56]), based on the cod recruitment index. (b–e) Spatio-temporal changes of the environmental factors most relevant for the recruitment are also shown with one year lag: (b) spring SST (°C), (c) *Calanus* ratio, (d) *C. helgolandicus* and (e) *Para/Pseudocalanus* spp. biomass (logmgDW/m^3^).

The results obtained by the model based on the entire North Sea showed that recruitment variability was mainly explained by SST, particularly during spring (i.e. 24.9±0.3% and 28.4±0.3% of explained deviance for annual and spring SST, respectively), rather than by the size of the adult stock (16.2±0.2%, Fig. 4b). Since 1998 spring SST increased following a south-north latitudinal gradient up to more than 1°C than in previous years, having a significantly negative effect on the density of cod recruits (Fig. 5b).

The results obtained by the model restricted to the polygons associated with the southern and central North Sea confirmed that spring SST had a higher impact on the recruitment variability than the size of the adult stock (respectively 19.8±0.3% and 6.6±0.2% of variance explained, Fig. 4c). Zooplankton biomass, in particular the *Calanus* ratio (which is an index of the relative dominance of *C. finmarchicus* on total *Calanus* spp. biomass) and the biomass of *C. helgolandicus* explained about 8% of the oscillations in cod recruits, the decrease of *C. finmarchicus* and the relative increase of *C. helgolandicus* being negatively correlated with the number of recruits (Fig. 5d). Total copepods and *Para/Pseudocalanus* spp. biomass had a significant positive effect on the recruitment, although they explained only 3–4% of the variability in the cross-validation procedure (Fig. 4e). The remaining zooplankton indices did not seem to be particularly relevant in relation to changes in cod recruitment (less than 2.6% - effect of *C. finmarchicus*).

Spatio-temporal variability in mature cod was strongly influenced by the fluctuations in annual SST (on average 32.8±1.4% of explained deviance, Fig. 4a). The variability of the adult stock was also significantly related (22.2±1.2%) to fishing mortality. The positive relation between adult stock and fishing mortality likely reflects a higher fishing pressure on greater stocks, rather than a positive effect of fishing mortality on adult biomass. Effect of temperature was also important for the *Calanus* ratio, which was negatively correlated (r_sp_ = −0.65, p-value<0.001) and strongly influenced (48% of explained deviance, Table 1) by spring SST (Fig. 4b,c). This was due to a strong negative influence of warmer temperatures on *C. finmarchicus* (Table 1) and on total *Calanus* biomass. Other indices were lower or not correlated with temperature (Table 1).

**Table 1:** Results of the polynomial LM models testing for the effect of spring SST on different indices of zooplankton.

Response variable	Expl. Dev. by Spring SST effect (%)
*Calanus* ratio	47.9
Biomass of *Calanus spp.*	19.6
Biomass of *C. finmarchicus*	31.7
Biomass of *C. helgolandicus*	7.6
Total biomass of zooplankton	6.8
Total biomass of copepods	*NS*
Biomass of *Para/Pseudocalanus spp.*	*NS*
Biomass of Euphausiids	14.5

Best models were obtained with a polynom of degree 2. All explained deviances (Expl. Dev.) were highly significant (p-value<0.1%, Chi-square test), except when non significant (*NS*). Residual degree of freedom = 246.

**Figure 6:**
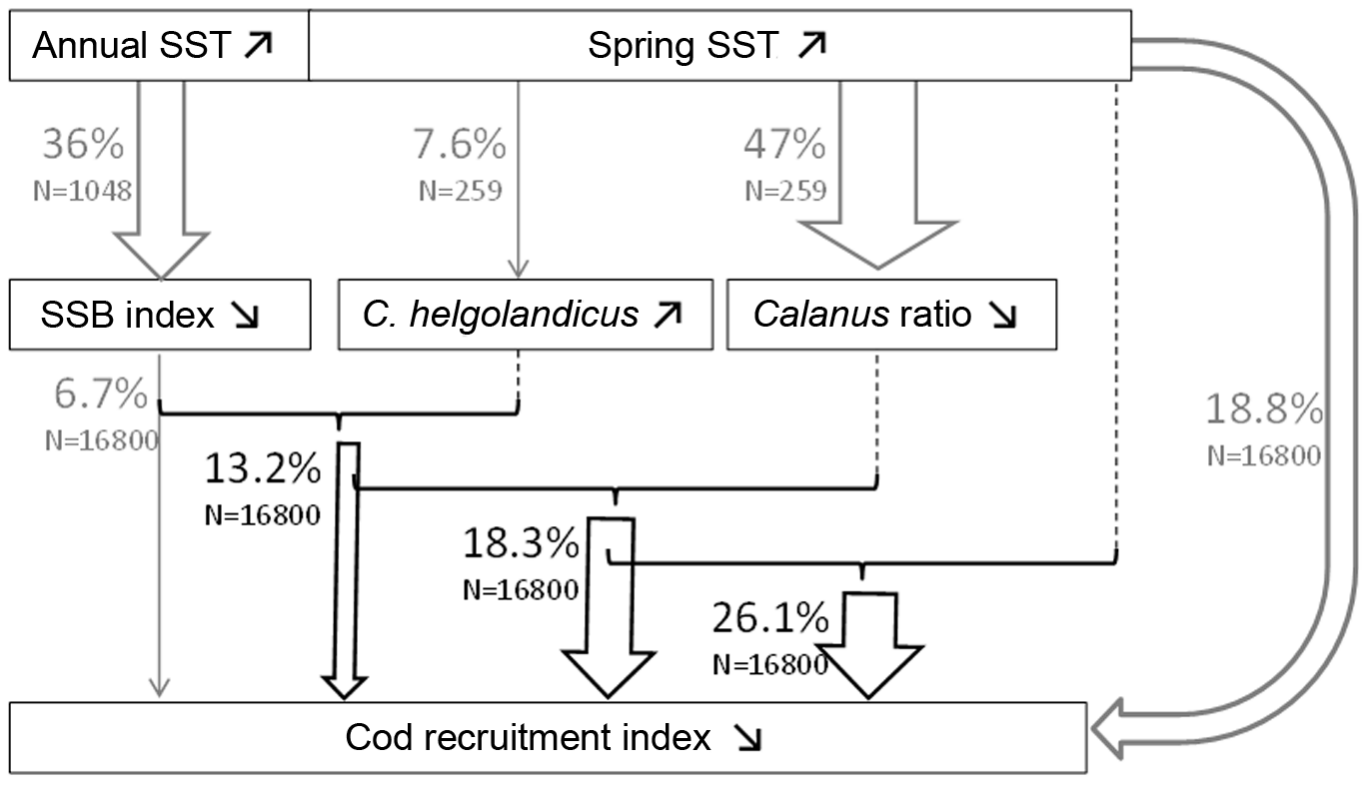
How the recruitment of North Sea cod is explained by temperature-driven processes. Conceptual model summarizing the influence of different environmental descriptors on the spatio-temporal variability of North Sea cod recruits. Note that arrows refer to the deviance explained by the model with one (grey) and several (black) covariates (see Table 2 for further details).

Spring temperature was identified as the main factor influencing recruitment variability (Table 2). Models not including SST as a covariate, even if including biomass indices directly correlated with temperature, explained less variability than the model with temperature only (3.3–6.7% against 18.8% of explained deviance, Table 2 and Fig. 6). The final model including the adult stock size, *C. helgolandicus* biomass and *Calanus* ratio, explained overall 18.3±1.1% of deviance (Table 2). Adding spring temperature further improved the model (26.1±1.2% of explained deviance), suggesting a remaining significant negative effect directly or indirectly related to temperature on cod recruitment (Table 2 and Fig. 6).

**Table 2:** Deviance explained by different environmental descriptors in the generalised additive recruitment models with one, two or more covariates.

	Mean explained deviance (% ± sd)*				Overall effect sign
Explanatory variables	First covariate effect	+ Second covariate effect	+ Third covariate effect	+ SST effect	
SSB index	**6,7±0,6**	/	/	/	+
*C. helgolandicus*	8,3±0,7	**6,4±1.1**	/	/	−
*Calanus* ratio	9,0±0,7	5,1±1.0	**5,9±1.4**	/	+
*Para/Pseudocalanus spp.*	4,0±0,6	4,6±1.1	NS	/	
Total Copepods	3,3±0,5	3,8±1.0	NS	/	
*C. finmarchicus*	NS	/	/	/	
Total zooplankton	NS	/	/	/	
*Calanus spp.*	NS	/	/	/	
Euphausids	NS	/	/	/	
Spring SST	18,8±0,9	15,6±1,2	12.0±1.4	**7,8±1.6**	−

The main descriptor selected at each step is indicated in bold (see methods for details). Uncertainty was computed using a cross-validation method coupled with bootstrap (see methods for details). ‘NS’ = Gain in explained deviance <3%; ‘/’ = Not tested because Not significant (NS) or already kept in a previous step.

*Form of model tested: gam(cod recruitment index ∼ Polygon+s(Var1, kmax = 2)+…+te(Var1, by = Polygon, kmax = 2)+…).

## Discussion

The present study aims at improving our understanding of the mechanisms underlying the recent decline of the North Sea cod stock, by taking the spatial component explicitly into account. Integrating three unique long-term spatially-resolved data sets (i.e. IBTS cod data, CPR zooplankton data and BADC SST data), we investigated the effects of changes in temperature, adult stock size and biomass of key zooplankton prey on cod recruitment. The spatio-temporal models here developed allowed testing correlation between changes in distribution of cod, zooplankton and temperature, in a new and complementary approach compared with previous studies mainly focusing on temporal variability, in which the spatial variability was not explicitly taken into account [17],or only explored in a few selected years [26]. Our method enabled us (i) to identify major shifts in cod spatio-temporal distribution and the associated uncertainty and (ii) to investigate correlation between environmental factors and cod recruitment by considering explicitly patterns of spatial heterogeneity [38]–[40]. By detecting main changes in the biotic and abiotic distributions, spatio-temporal models represents a promising first step towards the understanding and prediction of changes in the structure and functioning of ecosystems submitted to large-scale drivers such as climate change.

The length of the spatially-resolved time series analysed was limited by the data availability. Although CPR data are available in the North Sea since 1958, we investigated the period from 1973 onwards to allow comparison with IBTS fish data having a good spatial coverage. CPR data, relying on commercial ship routes, might display year to year heterogeneity in sampling coverage. Nonetheless, data were interpolated over a regular grid and averaged over 4 to 6 months, to ensure a consistent picture of zooplankton spatial distribution and a reduction of spatial auto-correlation. The CPR monitoring program provides the best available information on near-surface plankton biomasses at the temporal and spatial scales studied here. It does not report, though, for plankton variability throughout the whole water column.

A recent study highlighted heterogeneity in gears used for the IBTS dataset, in particular before 1983 [59], but this may have a non significant effect on our results. Indeed, calibration between different gears showed no evidence of bias for adult cod biomasses estimation. It showed a slight under-estimation of juvenile cods for some gears, but this only affects period 1974–1983, for a small proportion of gears (29%) and with a very limited effect (8% underestimation [59], Supplemental Material).

Our results showed a major drop in the recruitment of cod during the late 1990s and allowed us to assess whether and to what extent this change was related to changes in temperature, SSB and plankton prey (Fig. 6). In particular we identified: (i) a significant negative effect of warmer temperatures on recruitment, SSB and *Calanus* standing stock; (ii) a positive correlation between cod recruitment and both the relative dominance of *C. finmarchicus* and the adult stock size; (iii) a relative positive correlation between recruitment and biomass of *Para/Pseudocalanus* spp. All those correlations are supported by previous findings that have indicated temperatures <10°C and high abundance of favourite prey such as *C. finmarchicus* and *Pseudocalanus* spp., as suitable conditions for the recruitment of cod [17], [29], [35], [42].

Our best model, including the adult stock size, quantitative and qualitative *Calanus* descriptors and spring temperature, explained a quarter of the total variability of cod recruitment, which is valuable but hints at the implications of other phenomena. Other factors such as fishing mortality [2], stock structure [10], [60], larval drift [61] and competition or predation [19], [59], [62] could explain the remaining unexplained variability, but long-term spatio-temporal series needed to verify those potential pressures are not yet available.

### Direct vs Indirect Effects of Temperature

The sudden and long-lasting warming of the enclosed North Sea is associated with a more than 1°C increase in SST since the late 1990s. The increase in water temperature likely impacted the physiology of a large number of species, triggering multiple changes in the ecosystem [63], [64]. In agreement with previous studies [19], [22], [23], we showed that increasing temperature, particularly during the spring season, significantly compromised cod recruitment in recent years. Laboratory experiments showed that temperatures more than 9.6°C might directly affect cod recruitment success, reducing the fertilization and the normal development of the eggs [16]. Other direct effects include an earlier time of spawning, earlier egg hatching and a faster rate of larval development, leading to an earlier transition to exogenous first feeding and higher metabolic requirements [20], [24], [25]. The low availability of favourite plankton prey associated with the SST’s rise, may further compromise recruitment success. Indeed, with increasing temperatures, primary production changed in terms of composition, abundance and spring bloom timing, affecting in turn zooplankton productivity and the food available to fish larvae [40], [65]. Coupled physical and physiological models have shown a lower cod larval survival during warm years as compared to cold ones, probably due to a spatio-temporal mismatch between first-feeding cod larvae and their prey [26]. Thus, the indirect effects of temperature through bottom-up control on cod recruitment could be more important than the direct ones.

As a consequence of climate change cod, as well as other fish species [64], have contracted their distribution northwards to remain in suitable thermal habitats. The latitudinal shift in adult cod distribution, significantly related to the northwards displacement of the annual 10°C isotherm (results not shown), as already noted by Beaugrand et al. [35], could have possibly led to local failures in recruitment, particularly in the southernmost sub-stocks.

### Indirect Effect of Temperature through SSB

The significant decrease of adult cod observed in the North Sea since 2001 was anticipated by an abrupt drop in its recruitment. This was particularly the case in the south-eastern sub-stock (i.e. polygon A, Fig. 2a), which represented the southernmost subpopulation of cod in the Eastern Atlantic. The failure in recruitment was associated with the most significant temperature rise that has occurred since 1930 (results not shown). Cod recruits in the south-eastern North Sea, isolated by distance and oceanography [66], tend to reproduce in the area maintaining a genetically separate population [54], [56], [67]. The significant decrease of recruits preceding the drop of this sub-stock thus suggests that local depletion [67] rather than a northwards migration of the adults [64], [68] was the main cause of cod’s decrease in this North Sea region.

It has previously been hypothesized that the intensification of westerly winds associated with positive winter NAO conditions enhances the northwards transport of eggs and larvae, thus leading to a northwards shift of cod populations [68], [69]. Although this mechanism may have contributed to the depletion of the south-eastern sub-stock, it did not appear as the main cause of the sudden decline in cod recruitment during the late 1990s, as persistent positive NAO conditions were already recorded since the late 1980s. The results of the present study rather suggest that the recent failure in recruitment was mainly related to a significant change in environmental conditions associated with a sharp increase in SST. This confirms that the viability of a stock, with a structure weakened by decades of overfishing, greatly relies on recruitment success, hence on environmental conditions [9], [10].

The IBTS fishing survey data used here allowed the formation of a relevant picture of the spatial distribution of adult density, during or just before the spawning period, which is supposed to integrate the possible spatial impact of fishing activity on egg production. Due to the lack of spatially-resolved long-term data, the present analysis did not disentangle the influence of fishing mortality on the size of the adult stock from other environmental factors. Records of cod CPUE derived by British trawler landings indicate considerable spatio-temporal changes in trawling effort across the North Sea over the last century [70]. Indeed, as previously suggested [42], [71], during the 1990s increasing fishing mortality may have exacerbated the warming effect, particularly on the southern subpopulation [70]. This would explain the decline of adult density in the southern North Sea, and contribute to the drop in recruitment. Nonetheless, our analysis indicates that the adult stock size was not among the main factors correlated with the recent failure in cod recruitment. Moreover, a recent diagnosis of the fishing impact on European commercial fish stocks reveals that, despite a decrease in the fishing pressure over the last decade, assessed stocks by the ICES remained at low levels in the North Sea, possibly due to low recruitment success [72].

### Indirect Effect of Temperature through Bottom-up Control

Changes in the zooplankton community associated with increasing temperature, rather than the size of the adult stock, were correlated to the recent failure in cod recruitment [17], [29]. *Calanus* spp., the cold-temperate water species *C. finmarchicus* and the temperate water *C. helgolandicus* [17], dominated the copepod community in terms of biomass, particularly in the central and northern North Sea [31], [73]. The relative proportion of *C. finmarchicus* vs *C. helgolandicus* (i.e. *Calanus* ratio) appeared to be a good proxy of North Sea warming [74]. *Calanus* ratios around 0.5 (corresponding to equal abundance of the two *Calanus* species) were associated with an annual mean temperature of 10°C, which corresponds to the isotherm separating the cold Atlantic Polar biome and the warmer Westerly Winds biome [35]. Experimental work indicates 10°C as a critical threshold for different *Calanus* species, with *C. helgolandicus* developing faster than *C. finmarchicus* above 11°C [32]. Consequently, the *Calanus* ratio can indeed be considered as a good biological indicator of climatic shifts in the North Sea [74]. It also represents a good proxy to assess the quality of cod larval *Calanus* prey, as *C. helgolandicus* is less fat and less nutritious due to a lower level of fatty acids content than *C. finmarchicus* [75], [76].

Our method, considering the periods identified by the analysis of cod recruitment variability, did not allow the detection of a decline in the total biomass of *Calanus* spp, which could have affected cod recruitment in the late 1990s. On the other hand, the spatially-explicit analysis of total zooplankton biomass over the period 1958–2009 (Figure S1), pointed out that during the late 1990s, when *C. finmarchicus* was at its lowest abundance (not shown, [47]), the zooplankton standing stock was at the minimum. The decline of the biomass of *C. finmarchicus* started before the early 1970s and was only slightly mitigated by a rise in the biomass of *C. helgolandicus* since the late 1990s, mainly in the central and northern North Sea, where cod recruitment remained high. Hence, a decline in the quantity of *C. finmarchicus* prey may also be at the origin of the drop of cod recruitment.

In the south-eastern region, *Pseudocalanus* spp. is considered as an important prey for cod larvae [42]. Interestingly, the biomass of these potential prey and in turn of total copepods decreased significantly in the late 1990s particularly in the south-eastern region, where by contrast the biomass of total small zooplankton species (<2 mm length size) increased significantly from the late 1990s (results not shown). These significant changes in the zooplankton community may further have constrained cod larval survival.

## Conclusions

Due to decades of overexploitation, the North Sea cod stock has dramatically declined [2]. Despite a decrease in fishing pressure over the last decade [3], [77], no clear recovery of the North Sea cod stock has been observed, suggesting that other pressures are acting on this already weakened population [72]. The present study supports previous hypotheses that environmental changes may be related to poor fish recruitment [17], and adds a further step in the identification of main sources of variability of recruitment from a spatio-temporal angle. Climate change appears as one of the major drivers of the recent failure in cod recruitment, by acting directly on the biology of early life stages and indirectly on the quality and quantity of their zooplankton prey. Since the late 1990s, unfavourable thermal conditions and lack of favourite zooplankton prey have reduced the numbers of cod recruits particularly in the south-eastern North Sea, suggesting that local depletion rather than northwards migration of the adults was the main cause of the progressive decrease of North Sea cod in most recent years. Assuming that due to climate change the North Sea average temperature continues to rise, both direct and indirect effects of temperature will conspire to further reduce the cod stock, regardless of any management measure. According to Cabral et al. [77] the North Sea cod stock is exhibiting some signs of recovery, supposedly linked with the decrease of fishing pressure implemented since 2005. Future work, incorporating new data in regional recruitment models, will help to evaluate the potential influence of plankton species in this possible recovery. The development of spatio-temporal models, coupled with physical and physiological models [26], [61] validated by most recent observations, might further help to disentangle the main causal mechanisms involved in the fluctuations of cod recruitment. Including data on temperature and zooplankton would also improve estimation of recruits and adult biomass for the assessment of spawning stock biomass dynamics.

## Supporting Information

Figure S1 **Spatio-temporal variability of total zooplankton biomass.** Identification of major changes in the spatio-temporal variability of total zooplankton biomass (logmgDW/m^3^) between 1958 and 2009, based on March-September averages. Panels are described in the caption of Figure 2.
(TIF)
