# Supplementary figures and images for: Spatio-Temporal Variability of the North Sea Cod Recruitment in Relation to Temperature and Zooplankton
Source: PLoS One. 2014 Feb 13;9(2):e88447. doi: 10.1371/journal.pone.0088447 (PMC3923776; doi:10.1371/journal.pone.0088447)

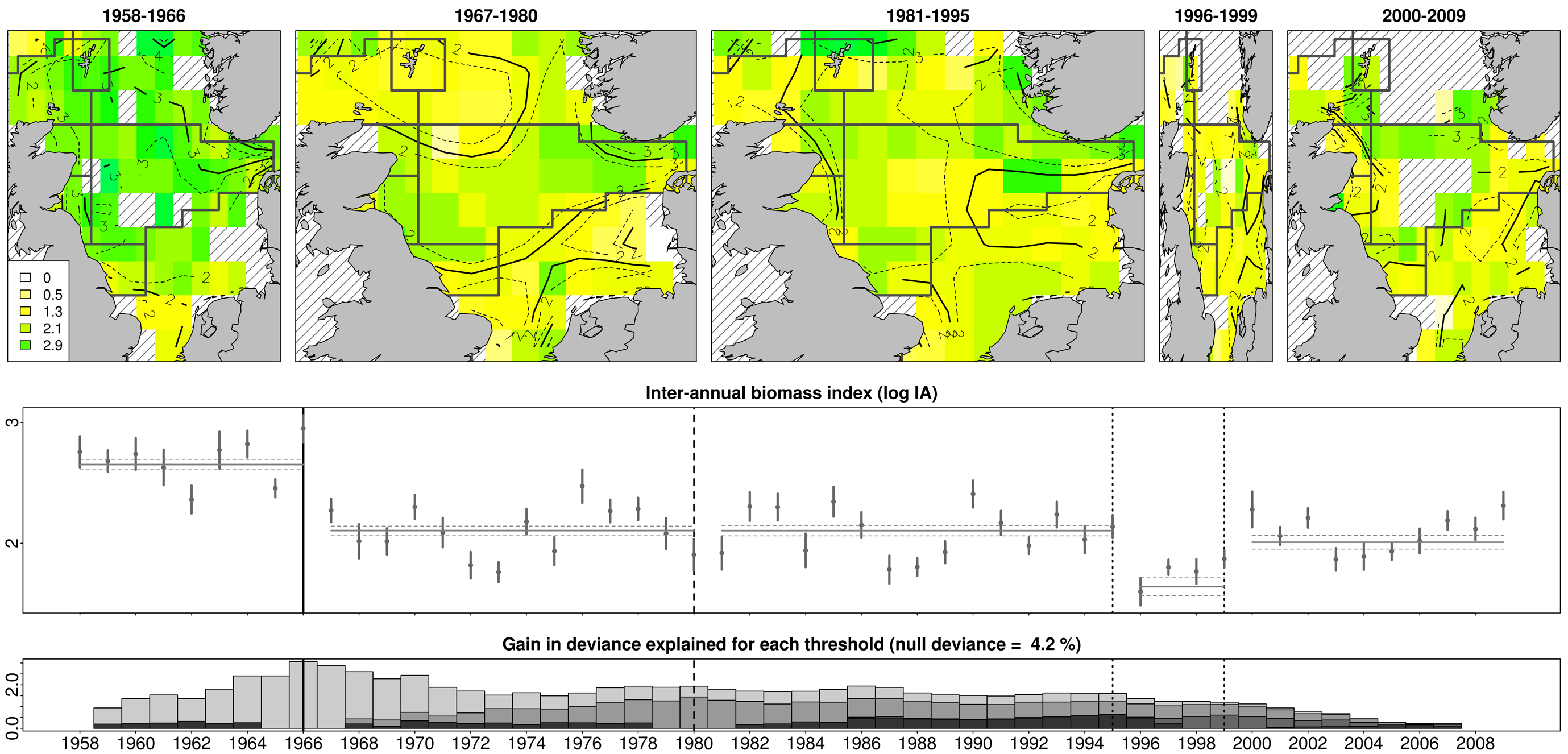

Supplement: Figure S1 — Spatio-temporal variability of total zooplankton biomass. Identification of major changes in the spatio-temporal variability of total zooplankton biomass (logmgDW/m3) between 1958 and 2009, based on March-September averages. Panels are described in the caption of Figure 2. (TIF) [file pone.0088447.s001.tif]
